# Systemic investigation of inetetamab in combination with small molecules to treat HER2-overexpressing breast and gastric cancers

**DOI:** 10.1515/biol-2022-0535

**Published:** 2023-01-10

**Authors:** Lan Deng, Le Zhao, Lifen Liu, Haomin Huang

**Affiliations:** R&D Department, Sunshine Guojian Pharmaceutical (Shanghai) Co. Ltd, a 3SBio Inc. Company, 399 Libing Road, Shanghai, 201203, China

**Keywords:** inetetamab/Cipterbin, trastuzumab/Herceptin, TKI, HER2, breast cancer, gastric cancer

## Abstract

Most patients with metastatic breast cancer or gastric cancer who are treated with trastuzumab, an anti-HER2 monoclonal antibody, become refractory to the drug within a year after the initiation of treatment. Although the combination of trastuzumab with pertuzumab produced synergetic effects in the treatment of HER2-overexpressing cancers, not all patients with HER2 overexpression benefited from the trastuzumab plus pertuzumab combination. To improve the clinical benefits of trastuzumab, we systemically investigated the combination of inetetamab (Cipterbin), an analog of trastuzumab, with a variety of small molecules, including tyrosine kinase inhibitors (TKIs) and chemotherapeutic agents *in vivo*. We showed that pan-TKIs-induced synergistic antitumor effects with inetetamab in the treatment of these two types of cancers and that adding chemotherapeutic agents to the existing TKI plus anti-HER2 monoclonal antibody combination strategies induced additional inhibitory effects, suggesting that such combination strategies may be choices for the treatment of these two tumors. Thus, combination therapies targeting distinct and broad pathways that are essential for tumor growth and survival can be effective for treating metastatic breast cancers and gastric cancers.

## 1 Introduction

Gastric cancer and breast cancer are two of the most common malignant cancer types worldwide. According to estimates from the International Agency for Research on Cancer, female breast cancer has surpassed lung cancer as the most commonly diagnosed cancer, with an estimated 2.3 million new cases in 2020. In women, breast cancer is the most commonly diagnosed cancer and the leading cause of cancer death [1]. Gastric cancer is the third-most common cause of cancer-related death after lung and colorectal cancers, there were 1,033,701 new cases of gastric cancer (representing 5.7% of all cancer cases diagnosed) and 782,685 deaths related to gastric cancer in 2018 [2,3].

The HER-2/neu oncogene encodes a 185-kDa transmembrane tyrosine kinase receptor that is upregulated in approximately 20% of breast cancers and 13–23% of gastric cancers [4,5]. HER2 gene amplification and protein overexpression play important roles in tumorigenesis. Accumulating evidence has revealed that HER2 overexpression is associated with higher tumor grade and a high risk of relapse and death [6]. Therapies with HER2-targeting monoclonal antibodies (mAbs), such as trastuzumab and pertuzumab, have shown promising clinical benefits in treating HER2- overexpressing cancers for decades [7,8,9]. Trastuzumab (Herceptin), a recombinant humanized anti-HER2 mAb developed by Roche/Genentech, is widely used in the first-line treatment of HER2-overexpressing metastatic breast cancer and has also exhibited clinical benefits in overall survival (OS) when used in the first-line treatment of HER2-overexpressing advanced gastric cancer [10,11]. In the clinic, an objective response rate (ORR) of 40% can be achieved in HER2-overexpressing metastatic breast cancer when trastuzumab is prescribed as a single agent. However, only 12–34% of patients with HER2 gene or protein upregulation responded to trastuzumab monotherapy, with a median duration of 9 months [10]. Generally, most of the patients who initially respond to trastuzumab develop resistance within 1 year [10]. Therefore, the combination of trastuzumab with other antitumor agents is a reasonable strategy to enhance the clinical outcome in treating HER2-overexpressing cancers.

Previous studies have reported that trastuzumab can be utilized to sensitize HER2‑overexpressing cancer cells to radiotherapy because it inhibits HER2 signaling pathways [12,13]. Other studies demonstrated that trastuzumab in combination with chemotherapeutic agents, such as paclitaxel or vinorelbine, and with another anti-HER2 mAb, pertuzumab, exhibited synergistic antitumor effects with an ORR > 60% [9,13]. As a result, the current standard of care for patients with HER2-positive breast cancer in the clinic is trastuzumab in combination with pertuzumab (Perjeta), another anti-HER2 mAb developed by Roche/Genentech, plus paclitaxel [14]. When used as the first-line treatment of gastric cancer, these therapies also substantially increase OS and progression-free survival in clinics [15]. However, although the combination of trastuzumab with pertuzumab produced synergetic effects in treating HER2-overexpressing cancer cells, not all patients with HER2-overexpressing cancers benefited from trastuzumab plus pertuzumab combination therapy [9,16].

To address the unmet need for new therapies to treat HER2-overexpressing breast and gastric cancers, we systemically investigated the combination strategies using inetetamab (Cipterbin) [17,18], a newly marketed anti-HER2 mAb developed by Sunshine Guojian Pharmaceutical Company, in combination with a set of small-molecule antitumor agents in xenograft models. We showed that inetetamab combined with small molecules, mainly tyrosine kinase inhibitors (TKIs), exhibited stronger antitumor effects than the small molecules or inetetamab alone or trastuzumab in combination with pertuzumab, indicating that inetetamab has synergistic antitumor effects when combined with the right small molecules. Our results highlight the importance of TKIs in combination with anti-HER2 mAbs, such as inetetamab, in treating patients with HER2-overexpressing gastric cancers or breast cancers refractory to trastuzumab and provide insight into the design of new combination therapies with anti-HER2 mAbs.

## 2 Materials and method

### 2.1 Cell culture

The cell lines used in this study were obtained from American Type Culture Collection unless otherwise noted. Cells were cultured in a 37°C incubator with 5% CO_2_ using standard cell culture methods and reagents, and JIMT-1 cells were cultured in DMEM (Thermo Fisher, USA, Cat# 10569010) with 10% FBS (Thermo fisher, USA, Cat# 10099141C) and 1% penicillin/streptomycin (Thermo Fisher，USA, Cat# 15140122). NCI-N87 and HCC1954 were cultured in RPMI-1640 medium (Thermo Fisher, USA, Cat# 31870074) with 10% FBS, 1% L-glutamine (Thermo fisher, USA, Cat# A2916801) and 1% penicillin/streptomycin. Pyrotinib (Cat# HY-104065B), lapatinib (Cat# HY-50898), neratinib (Cat# HY-32721), cyclophosphamide (CTX; Cat# HY-17420), and capecitabine (Cat# HY-B0016) were purchased from MedChemExpress (Shanghai, China). Pertuzumab (Lot: H0324B01) and trastuzumab (Lot: N3883) were purchased from Roche (Shanghai, China). Inetetamab (Lot: 302-118001) was obtained from Sunshine Guojian Pharmaceutical (Shanghai, China). The isotype control used in the study was an IgG1 noncognate mAb produced in house.

### 2.2 IgG1 mAb production

The construct expressing the HER2 noncognate mAb was generated using pcDNA3.4 vector (Thermo Fisher, Cat#A14697). Transient transfection was performed by co-transfection of expression vectors encoding a heavy chain or a light chain individually into FreeStyle™ HEK293-F cells (Thermo Fisher, Cat#A14697) using 1 µg/mL 25 kDa linear polyethylenimine (Polysciences, Inc., Cat#23966-1). One day after transfection, Valproic acid (Sigma, Cat#P4543-25G) was added to the cell culture at a final concentration of 3 mM. On day 2 post-transfection, medium comprising 10% GlutaMAX, 10% 400 g/L glucose, and 80% freestyle 293 medium was added to the cell culture at 10% of the total volume. Conditioned medium was collected 5–6 days after transient transfection. Antibodies in the culture media were purified by MabSelect SuRe affinity columns (GE Healthcare) on an AKTA Avant 25 fast protein liquid chromatography system. The columns were equilibrated with buffer A (25 mM sodium phosphate, 150 mM sodium chloride, pH = 7.0) prior to use. The culture media containing antibodies were then applied to the columns followed by elution with buffer B (100 mM sodium citrate, pH 3.5) to collect the desired proteins. The collected proteins were neutralized with 1 M Tris (pH 9.0), which were then dialyzed against phosphate-buffered saline. Finally, the purity of the samples was analyzed on a size exclusion chromatography-high performance liquid chromatography (SEC-HPLC), using 7.8 mm × 300 mm SEC columns (TSKgel G3000SWxL, Tosoh 08541) running with 200 mM sodium phosphate, pH 6.8 at 0.5 mL/min.

### 2.3 Tumor xenografts in mice

Cell line-derived xenograft models were established in 6–8 weeks old female CB-17 SCID mice or BALB/c nude mice by subcutaneous injection of 8 × 10^6^ JIMT-1 cells, 5 × 10^6^ NCI-N87 cells, or 5 × 10^6^ HCC1954 cells mixed with 50% Matrigel. When tumors had reached a volume of approximately 200−400 mm^3^, the animals were randomly divided into groups (*n* = 8/group) with comparable tumor sizes and treated as follows: antibodies were intraperitoneally administered twice a week; pyrotinib, CTX, lapatinib, capecitabine, and neratinib were orally administered daily five times a week. Tumor volume was measured by a caliper twice a week and calculated using the formula *V* = *LW* ^2^/2 (where *V* = volume, *L* = length, and *W* = width). The body weight of each animal was also measured twice a week. All animals were sacrificed after 5 weeks of treatment, and tumors were then excised and weighed individually.


**Ethical approval:** The research related to animal use has been complied with all the relevant national regulations and institutional policies for the care and use of animals, and were approved by the institutional IACUC of Sunshine Guojian Pharmaceutical (Shanghai) Co. Ltd, and performed under approved protocols (approval codes: AS-2020-006, AS-2020-054, AS-2020-011, and AS-2019-066).

### 2.4 Statistical analysis

Mouse xenograft data are presented as the mean value ± standard error. Statistical analysis was performed with GraphPad Prism 7 software and Excel. *p* values were calculated using a two-way ANOVA followed by Tukey’s multiple comparison test. For all tests, differences with *p* values <0.05 (*) were considered statistically significant.

## 3 Result

### 3.1 Inetetamab in combination with pyrotinib or CTX exhibited distinct antitumor effects in human breast cancer, JIMT-1 xenograft mouse model

**Figure 1:**
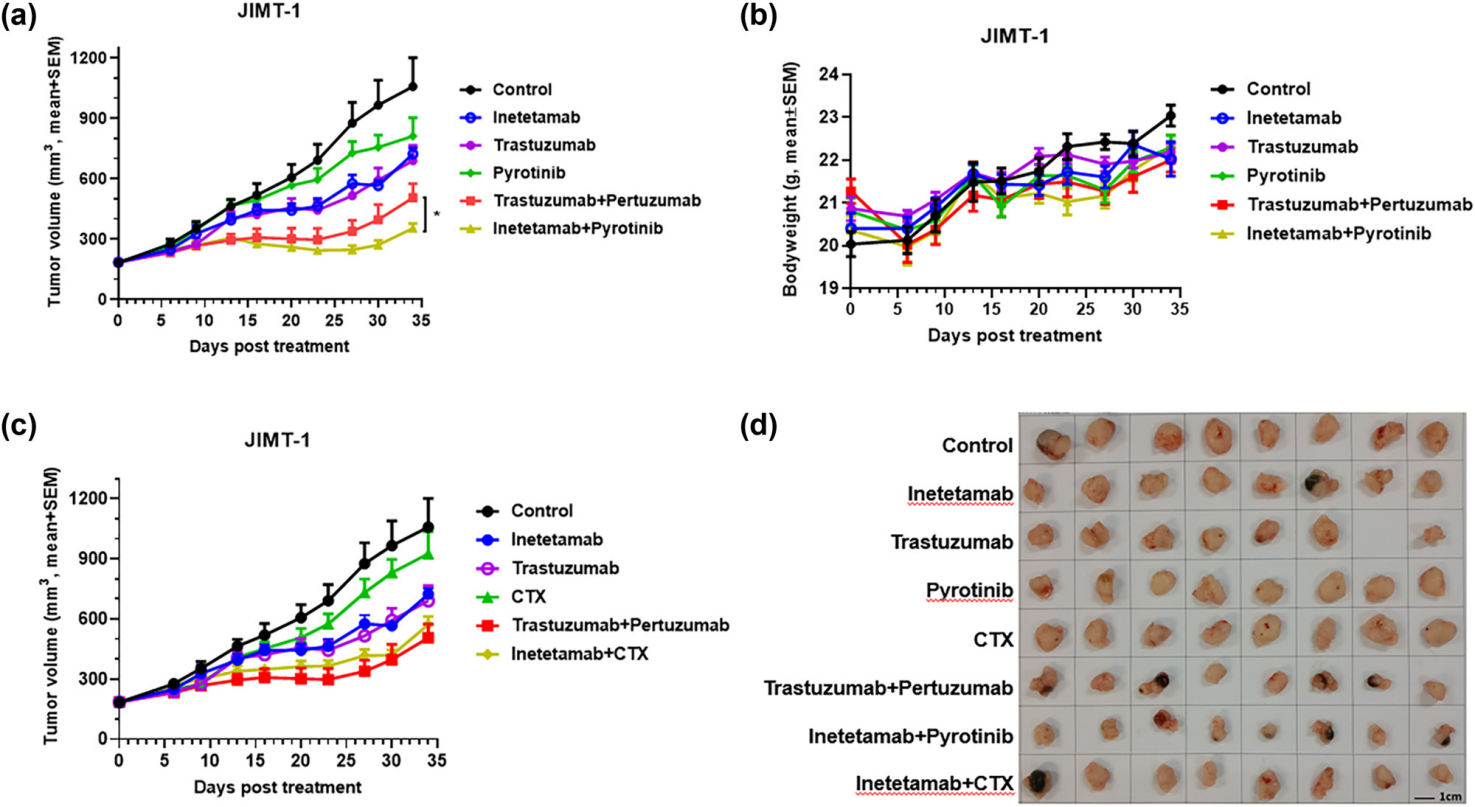
Inetetamab in combination with pyrotinib or CTX exhibited distinct antitumor effects in the JIMT-1 xenograft mouse model. (a) 20 mg/kg isotype control (black circle), 20 mg/kg inetetamab (blue circle), 20 mg/kg trastuzumab (purple circle), 20 mg/kg pyrotinib (green diamond), 20 mg/kg trastuzumab + 20 mg/kg pertuzumab (red square), and 20 mg/kg inetetamab + 20 mg/kg pyrotinib (yellow triangle) were i.p. injected into mice (*n* = 8) bearing JIMT-1 tumors. Tumor volumes (mm^3^) were measured at the indicated time points. Mean value ± SEM. *p < 0.05 by two-way ANOVA. (b) The mouse body weights of each treatment group were measured at the indicated time points. (c) 20 mg/kg isotype control (black circle), 20 mg/kg inetetamab (blue circle), 20 mg/kg trastuzumab (purple open circle), 10 mg/kg CTX (green triangle), 20 mg/kg trastuzumab + 20 mg/kg pertuzumab (red square), and 20 mg/kg inetetamab + 10 mg/kg CTX (yellow diamond) were i.p. injected into mice (*n* = 8) bearing JIMT-1 tumors. Tumor volumes (mm^3^) were measured at the indicated time points. (d) The photographs of tumors taken from mice in indicated groups.

To address the resistance to trastuzumab therapies, we first combined inetetamab with pyrotinib, an irreversible pan-HER receptor TKI targeting HER1, HER2, and HER4 [19,20], to treat JIMT-1 tumors in a xenograft mouse model. JIMT-1 is considered a trastuzumab-resistant cell line that was used for the development of pertuzumab by Genentech [21]. Inetetamab, a trastuzumab analog that has the exact F(ab’)2 of trastuzumab fused to a different IgG1 Fc allotype that differs from trastuzumab by two amino acids was recently approved by the Chinese National Medical Products Administration for marketing in China under the trade name of Cipterbin [17]. When used as a single agent, inetetamab or trastuzumab inhibited tumor growth to a limited degree, with a tumor growth inhibition (TGI) rate of approximately 40% for either trastuzumab or inetetamab on day 35 posttreatment. This TGI rate was slightly better than that of pyrotinib alone, which inhibited approximately 21% of tumor growth (Figure 1a). In contrast, the combination of inetetamab with pyrotinib exhibited much stronger tumor growth inhibition with a TGI rate of approximately 72%, significantly better than that of the two anti-HER2 mAbs (trastuzumab and pertuzumab) combined (*p* < 0.05), suggesting that pyrotinib may induce a stronger synergistic antitumor effect when combined with inetetamab than that of the current standard of care (trastuzumab plus pertuzumab) (Figure 1a). Importantly, no significant toxicity was seen across all treatment groups, since the animals from all groups did not lose body weight by the end of the study (Figure 1b).

Next we combined inetetamab with CTX, an alkylating cytotoxic drug commonly used for treating various cancers and used the combination to treat JIMT-1 tumors. In contrast to inetetamab combined with pyrotinib, inetetamab plus CTX did not outperform the combination of trastuzumab with pertuzumab, indicating that JIMT-1 cells may be more sensitive to TKI, such as pyrotinib, than to CTX combined with anti-HER2 mAbs (Figure 1c).

### 3.2 Inetetamab in combination with pyrotinib plus capecitabine exhibited superior antitumor effects in the HCC1954 xenograft mouse model

**Figure 2:**
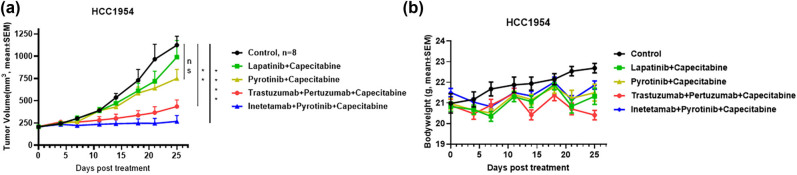
Inetetamab in combination with pyrotinib plus capecitabine exhibited superior antitumor effects in the HCC1954 xenograft mouse model. (a) 20 mg/kg isotype control (black circle), 100 mg/kg lapatinib + 200 mg/kg capecitabine (green square), 20 mg/kg pyrotinib + 200 mg/kg capecitabine (yellow triangle), 20 mg/kg trastuzumab + 20 mg/kg pertuzumab + 200 mg/kg capecitabine (red circle), and 20 mg/kg inetetamab + 20 mg/kg pyrotinib + 200 mg/kg capecitabine (blue triangle) were i.p. injected into mice (*n* = 8) bearing HCC1954 tumors. Tumor volumes (mm^3^) were measured at the indicated time points. As of 25th day posttreatment, the HCC1954 tumor appeared to be cystic with a maximum diameter of 20.49 mm. Mean value ± SEM. ** *p* < 0.01 and **** *p* < 0.0001 by two-way ANOVA. NS stands for “Not Significant.” (b) The mouse body weights of each treatment group were measured at the indicated time points.

Encouraged by the promising phase II study of pyrotinib in combination with capecitabine in HER2-positive metastatic breast cancer [22], we next determined the efficacy of the combination of inetetamab with pyrotinib plus capecitabine, a chemotherapeutic agent acting as a thymidylate synthase inhibitor, in treating HCC1954 tumors, another trastuzumab-resistant breast cancer model [23]. Both TKIs, lapatinib and pyrotinib, combined with capecitabine failed to show antitumor effects on HCC1954 tumors. However, inetetamab with pyrotinib plus capecitabine resulted in complete tumor growth inhibition (*p* < 0.0001 vs isotype control) and exhibited better antitumor activity than trastuzumab combined with pertuzumab plus capecitabine (*p* < 0.01 vs isotype control), although the difference between the two groups was not statistically significant (*p* = 0.8, Figure 2a). Importantly, inetetamab combined with pyrotinib plus capecitabine did not show signs of toxicity as the animal body weights were not reduced by the end of the study on Day 25 (Figure 2b). In contrast, trastuzumab combined with pertuzumab plus capecitabine induced a noticeable drop in the animal body weights on day 25 posttreatment, indicating that inetetamab combined with pyrotinib plus capecitabine may be a better strategy than two anti-HER2 mAbs plus capecitabine (Figure 2a).

### 3.3 Neratinib in combination with inetetamab exhibited superior antitumor effects in human gastric cancer, NCI-N87 xenograft mouse model

**Figure 3:**
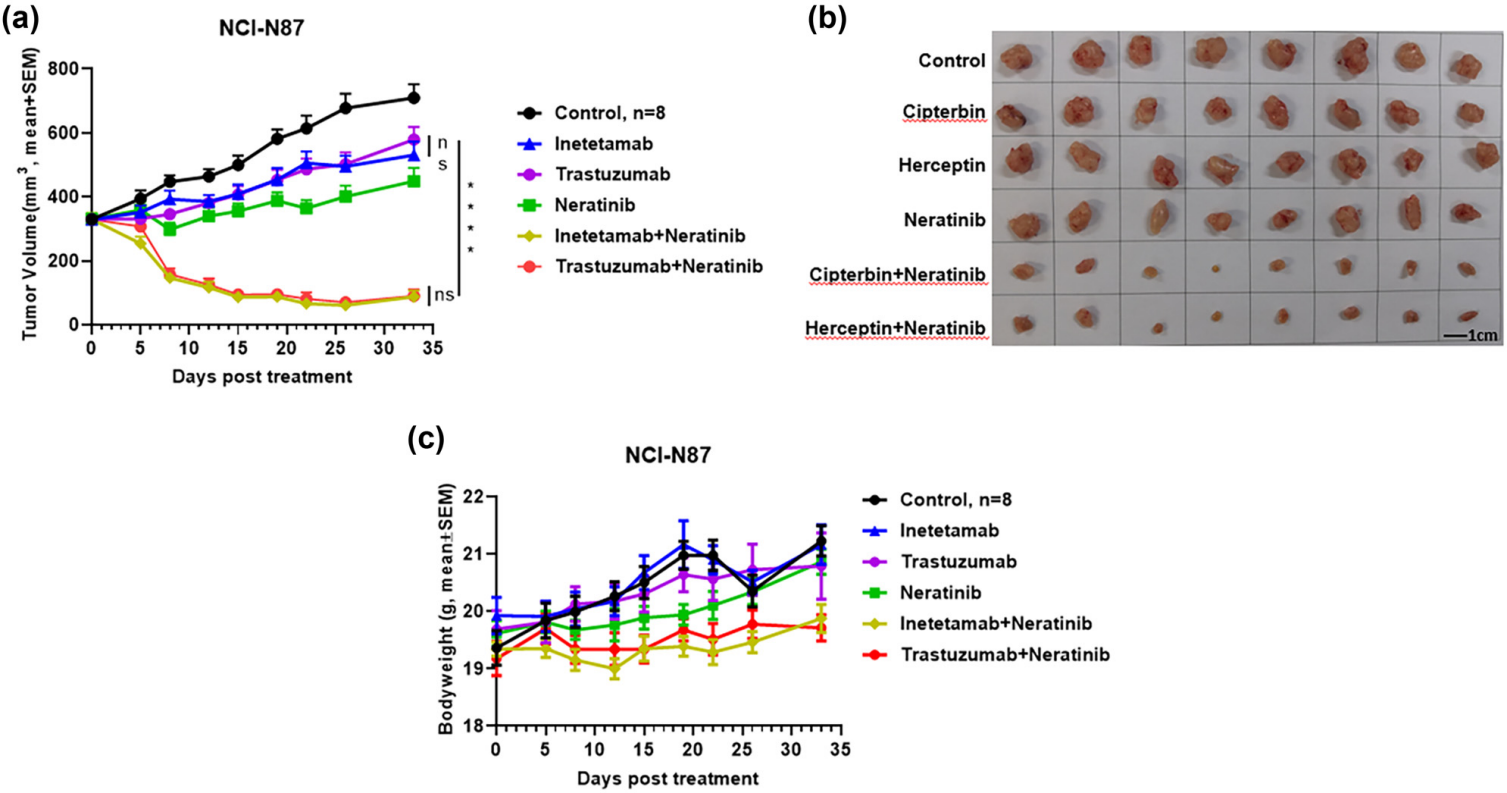
Neratinib in combination with inetetamab exhibited superior antitumor effects in the NCI-N87 xenograft mouse model. (a) 20 mg/kg isotype control (black circle), 20 mg/kg inetetamab (blue triangle), 20 mg/kg trastuzumab (purple circle), 10 mg/kg neratinib (green square), 20 mg/kg inetetamab + 10 mg/kg neratinib (yellow diamond), and 20 mg/kg trastuzumab + 10 mg/kg neratinib (red circle) were i.p. injected into mice (*n* = 8) bearing NCI-N87 tumors. Tumor volumes (mm^3^) were measured at the indicated time points. Mean value ± SEM. **** *p* < 0.0001 by two-way ANOVA. NS stands for “Not Significant.” (b) The photographs of tumors taken from mice in the indicated groups. (c) The mouse body weights of each treatment group were measured at the indicated time points.

To date, trastuzumab is still the only anti-HER2 mAb therapy available for gastric cancer [24], partly because several treatment strategies developed in the past that showed promise in the treatment of breast cancers failed to show clinical benefits in treating gastric cancer [25]. We next tested a new combination strategy of anti-HER2 mAbs combined with neratinib, a TKI with pan-HER inhibitory activities, in an NCI-N87 xenograft model. Trastuzumab or inetetamab combined with neratinib resulted in similar dramatic tumor regression despite initial tumor sizes as large as approximately 350 mm^3^ (*p* < 0.0001 vs trastuzumab/inetetamab). In contrast, neratinib and the two anti-HER2 mAbs (trastuzumab and inetetamab) alone exhibited moderate antitumor activities with TGI rates of 41, 27, and 26%, respectively, indicating that neratinib has the potential to provide additive antitumor activities to anti-HER2 mAb therapies (Figure 3a and b). Importantly, despite the potent antitumor efficacy, neither the combinations nor neratinib or the mAbs alone showed noticeable toxicities in animals, as no animals from any group showed a reduction in bodyweights (Figure 3c).

### 3.4 Inetetamab in combination with lapatinib or capecitabine exhibited superior antitumor effects in the NCI-N87 xenograft mouse model

**Figure 4:**
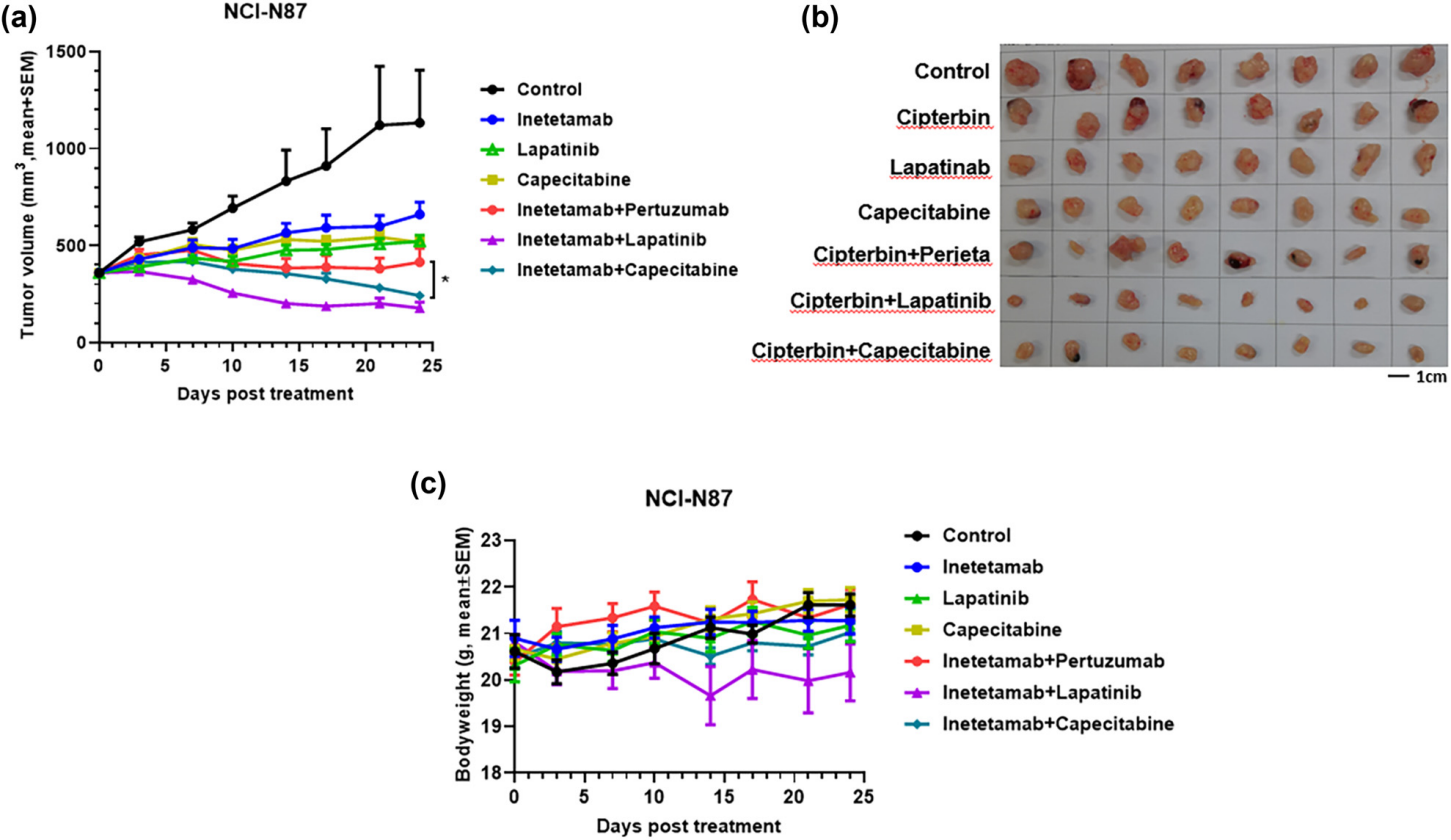
Inetetamab in combination with lapatinib or capecitabine exhibited superior antitumor effects in the NCI-N87 xenograft mouse model. (a) 20 mg/kg isotype control (black circle), 20 mg/kg inetetamab (blue circle), 100 mg/kg lapatinib (green triangle), 200 mg/kg capecitabine (yellow square), 20 mg/kg inetetamab + 20 mg/kg pertuzumab (red circle), 20 mg/kg inetetamab + 100 mg/kg lapatinib (purple triangle), and 20 mg/kg inetetamab + 100 mg/kg capecitabine (cyan diamond) were i.p. injected into mice (*n* = 8) bearing NCI-N87 tumors. Tumor volumes (mm^3^) were measured at the indicated time points. Mean value ± SEM. *p < 0.05 by two-way ANOVA. (b) The photographs of tumors taken from mice in indicated groups. (c) The mouse body weights of each treatment group were measured at the indicated time points.

**Figure A1:**
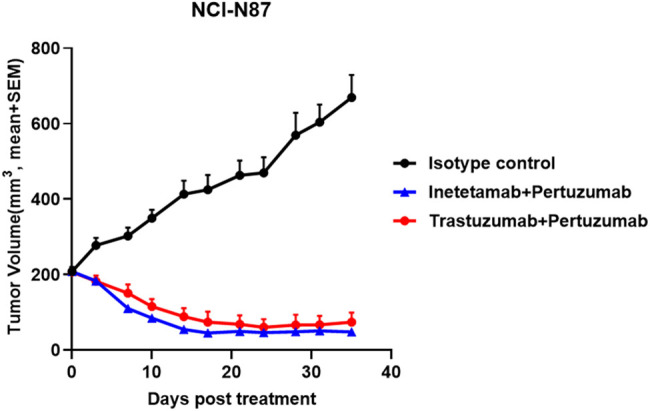
Inetetamab and trastuzumab are equally effective in inhibition of NCI-N87 growth when combined with pertuzumab in the NCI-N87 xenograft mouse model. 20 mg/kg isotype control (black circle), 20 mg/kg inetetamab + 20 mg/kg pertuzumab (blue triangle), 20 mg/kg trastuzumab + 20 mg/kg pertuzumab (red triangle) were i.p. injected into mice (*n* = 8) bearing NCI-N87 tumors. Tumor volumes (mm^3^) were measured at the indicated time points.

Previous studies demonstrated the effective antitumor activities of the triple combination of trastuzumab with capecitabine plus lapatinib, a dual TKI that targets EGFR and HER2 [26]. We were, therefore, interested in determining which combination (the two small molecules with distinct mechanisms of action combined separately with the anti-HER2 mAb) induced the largest synergistic effect. Given that inetetamab differs from trastuzumab only in the Fc region, as expected, they are equally effective in inhibition of NCI-N87 growth when combined with pertuzumab in our previous study (Figure A1) as well as when used as a single agent throughout the current study in various models (Figures 1(a and c) and 3a), we only showed inetetamab and inetetamab plus pertuzumab instead of trastuzumab or trastuzumab plus pertuzumab. Lapatinib combined with inetetamab induced significantly stronger tumor regression (TGI = 82%) than inetetamab (TGI = 47%) or lapatinib (TGI = 55%) alone, as well as inetetamab plus pertuzumab, which had a TGI rate of 66% (*p* < 0.05, Figure 4a and b). Similarly, capecitabine, the chemotherapeutic agent, combined with inetetamab also resulted in potent tumor regression comparable to that of inetetamab plus lapatinib by day 25 posttreatment (Figure 4a and b), suggesting that regardless of antitumor mechanism, with one inhibiting the cell cycle and the other targeting growth signaling pathways, the two agents exhibited synergistic antitumor effects similar to those of anti-HER2 mAb therapy in the treatment of NCI-N87 tumors. Of note, all these combination therapies again showed manageable toxicities, as the animal body weights barely changed throughout the study (Figure 4c).

## 4 Discussion

In this study, we tested the antitumor efficacies of inetetamab in combination with various small molecules, including chemotherapeutic agents and TKIs *in vivo*. Because most of these agents with distinct mechanisms of action have been studied individually in an animal model elsewhere, and because it is not uncommon that the data from mouse models cannot be directly applied to applications in the clinic due to the vast differences in the two species, we selected our *in vivo* doses for a specific agent based on the published animal studies, instead of their doses, if available, in clinical trials [27,28,29]. As a result, we confirmed that the small molecule antitumor agents were able to provide synergistic antitumor effects when combined with inetetamab. Importantly, combinations of these small molecules with anti-HER2 mAbs not only resulted in stronger tumor growth inhibition than monotherapy with individual small molecules or mAbs but also exhibited better antitumor effects than the current standard of care (trastuzumab combined with pertuzumab).

Strategies combining with trastuzumab with chemotherapeutic agents have shown better therapeutic outcomes than chemotherapy alone in HER2-positive cancers, resulting in higher pathological complete response rates and lower risks of disease relapse and death [30,31]. CTX is an alkylating cytotoxic chemotherapeutic agent with a broad anticancer spectrum. When used as a single agent, CTX did not induce significant tumor growth inhibition in the trastuzumab-resistant JIMT-1 model. However, when combined with inetetamab, the combination exhibited superior antitumor activity on par with that of trastuzumab combined with pertuzumab. Trastuzumab and pertuzumab recognize different epitopes on the HER2 extracellular domain (ECD) and presumably have complementary mechanisms, which might account for the better clinical outcomes of trastuzumab-refractory patients when these agents are given together [32,33]. As a result, killing of tumors through CTX-mediated DNA damage may provide a complementary force to overcome the resistance of tumors to anti-HER2 mAbs. Interestingly, pyrotinib combined with inetetamab resulted in significantly stronger tumor growth inhibition than trastuzumab combined with pertuzumab in the treatment of JIMT-1 tumors (Figure 1a). Pyrotinib is a pan-HER TKI, that inhibits EGFR, HER2, and HER4 [19]. These results suggest that broad inhibition of tyrosine kinase receptor-mediated growth and survival signaling might have better synergy with anti-HER2 mAb therapies than inhibition induced by DNA-damage.

Encouraged by the positive result of a phase II trial in which pyrotinib was used in combination with capecitabine in the treatment of HER2-positive metastatic breast cancer [22], we reasoned that the combination may be able to successfully counteract tumor resistance to anti-HER2 mAbs when combined with an anti-HER2 mAb. Indeed, the combination of inetetamab and pyrotinib plus capecitabine exhibited significantly stronger tumor growth inhibition than capecitabine plus either of the two pan-HER inhibitors, pyrotinib and lapatinib, in the trastuzumab-resistant HCC1954 model. Intriguingly, although statistical significance was not reached, the combination of inetetamab with pyrotinib plus capecitabine was able to inhibit HCC1954 tumor growth completely and thus appeared to be more effective in inhibiting HCC1954 growth than trastuzumab combined with pertuzumab plus capecitabine (Figure 2a). It is plausible that further optimization of doses and dosing schedules for the two triple combination strategies may eventually be able to result in significant difference between the two groups, considering that pyrotinib is a pan-HER inhibitor, that simultaneously blocks the signaling of three HER family members, whereas pertuzumab specifically binds and inhibits only HER2, and that the combination of inetetamab with pyrotinib resulted in stronger inhibition of JIMT-1 tumor growth than the combination of trastuzumab with pertuzumab (Figure 1a).

Mounting evidence has shown that treatments with anti-HER2 mAbs that are effective in breast cancers may not be effective in gastric cancers [25], emphasizing the difference between the two cancers and the need for distinct strategies for their treatment. Recently, Yoshioka and colleagues showed that HER2-positive gastric cancers are sensitive to pan-HER inhibitors, such as neratinib and lapatinib [34], suggesting that combing neratinib with an anti-HER2 mAb may be a better therapeutic strategy for the treatment of HER2-positive gastric cancer. Indeed, when we combined neratinib with inetetamab or trastuzumab, the combinations exhibited superior suppression of NCI-N87 tumor growth with TGI rates of >90%, whereas as a single agent, inetetamab, trastuzumab, or Neratinib showed a relatively low level of suppression with an average TGI rates of far less than 50% (Figure 3a). Similarly, lapatinib also significantly improved inetetamab antitumor efficacy, resulting in a better antitumor outcome than inetetamab combined with pertuzumab (Figure 4a). Intriguingly, although there was no significant difference between inetetamab plus lapatinib and inetetamab plus capecitabine, in terms of antitumor activity, the lapatinib combination seemed to act slightly faster than the capecitabine combination. These results support the idea that simultaneous inhibition of another tumor growth and survival dependent pathway in addition to HER2 blockade by mAbs is a promising strategy to treat gastric cancer. Nevertheless, further evaluations of more cancer lines, such as SK-BR-3 or AGS, and dosing schedules, including optimized concentrations, and dosing frequencies, are needed to better support the clinical designs. In addition, since mice did not die at the current experimental conditions and before the humane endpoints for euthanasia were reached, survival studies could not be performed. Another limitation of the present study is the lack of in-depth analysis of toxicity and pharmacodynamics (PD) in addition to the animal body weight measurement. Thus, further analysis of toxicities and PD in these animal models, including blood chemistry and kidney parameters, is necessary before testing in humans.

In general, the anti-HER2 mAb specifically binds to the ECD of HER2 and thus confines its inhibitory effects to the receptor-related signaling transduction, which can be exploited by tumors to become resistant through receptor mutagenesis or changes in compensatory pathways. By contrast, small molecule TKIs not only can readily move into cells, but also inhibits multiple intracellular targets of a variety of signaling pathways that may play roles in compensating for the loss of HER2 signaling. Indeed, crosstalk between insulin-like growth factor I (IGF-I) and HER2 plays an important role in the development of trastuzumab resistance and can be prevented by lapatinib-mediated IGF-I inhibition [35]. Similarly, nuclear localization of HER4 induces acquired resistance to trastuzumab, which can be counteracted by neratinib [36]. Thus, blocking pathways compensatory to HER2 signaling with TKIs may be beneficial in reversing the resistance.

In summary, we systemically analyzed the activities of various TKIs and chemotherapeutic agents in combination with inetetamab in the treatment of gastric tumors and breast tumors refractory to trastuzumab, to provide insight into the design of combination therapies. We showed that the pan-TKIs indeed induced synergistic antitumor effects in combination with anti-HER2 mAbs (inetetamab or trastuzumab) in the treatment of the two types of cancers. These results are most likely because in addition to the specific HER2 blockade mediated by the antibodies, the pan-TKIs provided additive and broader inhibitory effects on multiple signaling pathways that are required for tumor growth and survival. In line with this, it is plausible that adding additional agents to inhibit tumor growth with distinct modes of action from TKIs, such as chemotherapeutic agents, to existing combination strategies with TKIs plus anti-HER2 mAbs may offer more choices to treat these two aggressive tumors. Thus, combination therapies targeting distinct and broad pathways that are essential for tumor growth and survival can be effective for hard-to-treat cancers.
